# High-throughput sequencing reveals the structure and metabolic resilience of desert microbiome confronting climate change

**DOI:** 10.3389/fpls.2024.1294173

**Published:** 2024-03-05

**Authors:** Walaa K. Mousa, Tareq Abu-Izneid, Ahmed Salah-Tantawy

**Affiliations:** ^1^ College of Pharmacy, Al Ain University, Abu Dhabi, United Arab Emirates; ^2^ Al Ain University (AAU) Health and Biomedical Research Center, Al Ain University, Abu Dhabi, United Arab Emirates; ^3^ College of Pharmacy, Mansoura University, Mansoura, Egypt; ^4^ Institute of Analytical and Environmental Sciences, College of Nuclear Science, National Tsing Hua University, Hsinchu, Taiwan; ^5^ Department of Zoology, Marine Science Division, College of Science, Al-Azhar University, Assiut, Egypt

**Keywords:** desert microbiota, high-throughput sequencing, metagenomic, functional prediction, Arabian Peninsula, climate change

## Abstract

**Introduction:**

Desert ecosystems harbor a unique microbial diversity that is crucial for ecological stability and biogeochemical cycles. An in-depth understanding of the biodiversity, compositions, and functions of these microbial communities is imperative to navigate global changes and confront potential threats and opportunities applicable to agricultural ecosystems amid climate change.

**Methods:**

This study explores microbial communities in the rhizosphere and endosphere of desert plants native to the Arabian Peninsula using next-generation sequencing of the 16S rRNA gene (V3-V4 hypervariable region).

**Results:**

Our results reveal that each microbial community has a diverse and unique microbial composition. Based on alpha and beta diversity indices, the rhizosphere microbiome is significantly diverse and richer in microbial taxa compared to the endosphere. The data reveals a shift towards fast-growing microbes with active metabolism, involvement in nutrient cycling, nitrogen fixation, and defense pathways. Our data reveals the presence of habitat-specific microbial communities in the desert, highlighting their remarkable resilience and adaptability to extreme environmental conditions. Notably, we observed the existence of radiation-resistant microbes such as Deinococcus radiotolerans, Kocuria sp., and Rubrobacter radiotolerans which can tolerate high levels of ionizing radiation. Additionally, examples of microbes exhibiting tolerance to challenging conditions include Nocardioides halotolerans, thriving in high-salinity environments, and hyperthermophilic microbes such as Quasibacillus thermotolerans. Moreover, functional analysis reveals enrichment in chaperon biosynthesis pathways associated with correct protein folding under heat stress conditions.

**Discussion:**

Our research sheds light on the unique diversity of desert microbes and underscores their potential applications to increase the resilience of agriculture ecosystems, offering a promising strategy to fortify crops against the challenges posed by climate change, ultimately supporting sustainable food production for our ever-expanding global population.

## 1 Introduction

Desert ecosystems, despite being vital reservoirs of microbial diversity, have received limited attention in research (Alsharif et al., 2020; Weber et al., 2015). These microorganisms play a substantial role in maintaining ecological stability and influencing biogeochemical cycles within their environments. A comprehensive understanding of the biodiversity, compositions, and functions of desert microbial communities is crucial to gaining insights into global changes and for identifying potential threats and opportunities relevant to agricultural ecosystems in the context of climate change. There is growing interest in microbes that survive extreme and harsh environmental conditions and their potential applications to confront the consequences of climate change (Malhi et al., 2020; Matthews et al., 2017).

Microbes represent the oldest form of life with their metabolic versatility that enables adaptation to diverse ecosystems including the extremes. Some of the striking examples of bacterial resilience include 1) the genus *Aquifex*, which can survive temperatures exceeding 200 degrees Fahrenheit in hot springs (Mamat et al., 2009); 2) the *Thermococcus* genus, which can survive both extreme temperature and energy deprivation (Price et al., 2015); 3) the *Halobacterium* that survives 10 times more salt than found in seawater (Oren, 2008); and 4) the *Deinococcus radiodurans* bacterium that can survive a 15,000 gray dose of radiation, given that only 10 grays are sufficient to kill an adult human. Interestingly *D. radiodurans* is listed in the Guinness book as the world’s toughest bacterium (Krisko and Radman, 2013). Among the resilient microbes are those that survive and thrive in the harsh environment of the desert. Desert microbes encounter multitudes of environmental challenges and have evolved unique metabolic capabilities to tolerate stressful conditions and support the growth and resilience of their host plants (Thakur et al., 2022). Microbes in the desert are central to energy and nutrient cycling, the survival of desert plants, and the stability of the entire ecosystem (Leung et al., 2020). These microbes have already evolved to tolerate biotic and abiotic stress such as extreme heat while supporting the growth of their host plants, and could be used to compensate for missing species and function in other agricultural ecosystems—hence supporting sustainable food production for a growing global population amid climate change.

Desert landscapes span about one-third of the Earth’s total land area. Notable among these are the Sahara in North Africa (Goudie, 2009), the Gobi in North China (Rosen et al., 2019), the Thar in North India (Rao et al., 2016), and the Arabian Peninsula in the Middle East (Edgell, 2006). Part of the Arabian Peninsula is the UAE desert which represents a rich, diverse ecosystem that has not received adequate attention regarding its microbiome (Feulner, 2023; Alyammahi et al., 2023). The UAE desert is exposed to harsh weather conditions such as intense solar radiation, strong winds, enrichment in salts, sacrality of nitrogen and organic matter, and drought (Feulner, 2023). These conditions exert selection pressure on the microbial communities to select traits required to adapt and support the growth of their host plants. Here we applied the eDNA metagenomic approach to study the microbial composition and annotate metabolic pathways associated with rhizosphere and endosphere microbial communities of four desert plants native to the part of the Arabian Peninsula located in the UAE. Exploring the intricate microbial diversity and their associated functions within these desert environments presents unparalleled opportunities for uncovering distinctive capabilities and potential applications (Alsharif et al., 2020).

## 2 Methods

### 2.1 Sample collection

In this study, we selected four desert plants native to the UAE desert, namely *Halocnemum strobilaceum* (HS), *Panicum turgidum* (PT, known as the dessert grass), *Haloxylon persicum* (HP), and *Arnebia hispidissima* (AH, known as Arabian Primrose). Representative pictures of the plants are shown in **Supplementary Figure 1**. The inclusion criteria included being a native plant to the Arabian Peninsula desert near the UAE and being collected from different locations with diverse soil properties. In total, we collected 40 samples, 20 from the rhizosphere (soil attached to the root surface and in proximity) and 20 from inside the root tissues of the studied plants. We collected each plant from five adjacent location**s**, and GPS coordinates are provided in **Supplementary Table 1**. HS is collected from Torripsamments soil near Dubai city (25°02’38”N: 25°08’22”N, 55°29’01”E: 55°39’59”E). Torripsamments is the most extensive soil in the UAE covering 75% of the UAE and is formed of wind-borne sands on dunes and sand sheets (Shahid et al., 2014b; Shahid et al., 2014a; Shahid et al., 2014b). PT is collected from a Haplocalcids soil near Al Ain City (24°01’54”N: 24°05’11”N, 55°47’25”E: 55°49’54”E). Haplocalcids have a sandy and loamy texture and cover 1.7% of the UAE (Shahid et al., 2014b; Shahid et al., 2014a). HP is collected from Haplocambids soil near the As Salhiyyah area (25°32’54”N: 25°40’22”N, 55°53’23”E: 55°59’11”E). Haplocambids is a loamy soil which covers only 0.2% of the UAE (Shahid et al., 2014b; Shahid et al., 2014a). AH is collected from Torriorthents soil near Al Ain city (24°11’22”N: 24°12’21”N, 55°55’52”E: 55°57’27”E). Torriorthents is a dry salty soil and is located adjacent to mountains and is mostly sandy and loamy and covers 0.9% of the UAE (Shahid et al., 2014b; Shahid et al., 2014a). Samples were collected in October 2022 (the average day temperature is 34-40 °C, and chances of precipitation are low). Samples were kept in a sterile container, transported in an ice box, and stored at -80 °C for further processing. Next-generation sequencing and bioinformatic analysis have been conducted on pooled groups, each is composed of five subsamples.

### 2.2 DNA extraction from soil samples (the rhizosphere microbial communities)

To extract DNA from the rhizosphere, the attached soil layer is carefully removed and combined with the remaining soil collected from the rhizosphere zone, situated within a few millimeters of the plant (Gregory, 2006). DNA was extracted from soil samples using the NucleoSpin Soil Kit (Macherey-Nagel, Germany). The purity of the isolated DNA was assessed by electrophoresis using 1% agarose gel. The extracted DNA was then quantified using a NanoDrop TM Spectrophotometer (ThermoFisher Scientific, Waltham, MA, USA), and labeled.

### 2.3 DNA extraction from plant samples (The endosphere microbial communities)

The roots were surface sterilized to remove bacteria attached to the outer surface of the roots. Surface sterilization was conducted according to a previously described protocol with modifications (Coombs and Franco, 2003). Briefly, roots were immersed in autoclaved, distilled water and sonicated for 5 min to remove adherent soil particles. Then roots were soaked in 95% ethanol for 3 min followed by a sterile water rinse and this step was repeated twice. Thereafter, roots were subjected to a 5-minute wash in a 3% Sodium hypochlorite solution followed by washing with sterile water. The effectiveness of the surface sterilization process was evaluated by gently rolling the surface-sterilized root onto the tryptic soy agar medium under aseptic conditions. Subsequently, the samples were placed in an incubator at 25°C and 37°C for 3 days and checked for no bacterial growth before proceeding to the next step of DNA extraction, and amplicon profiling. Root tissues were ground in liquid nitrogen then DNA was subsequently extracted using the chloroform-isoamyl alcohol (the Edwards method) following the previously described protocol (Edwards et al., 1991; Tamari et al., 2013). The extracted DNA was subjected to agarose gel electrophoresis (1%) to check purity and then quantified using a NanoDropTM Spectrophotometer (TermoFisher Scientific, Waltham, MA, USA) followed by DNA labeling.

### 2.4 DNA amplification and next-generation sequencing

The hypervariable V3-V4 region of the 16S rRNA was amplified using the universal primer set 341F/785R (Klindworth et al., 2013). Previous studies showed that this primer pair exhibits the widest coverage of the domain bacteria with up to 96.1% without exhibiting a significant bias towards most of the bacterial species which makes it suitable for taxonomical classification of both plant and soil-associated bacteria (Thijs et al., 2017; Fadeev et al., 2021). In addition, previous reports show that these primers give reproducible results which are characterized by phylogenetic richness and higher diversity (Thijs et al., 2017; Fadeev et al., 2021). Both forward and reverse primers were tagged with Illumina adapters, pads, and linker sequences. Each 50 μL PCR reaction mixture containing 30 ng of template DNA, PCR primers, and PCR master mix (Taq DNA Polymerase, dNTPs, 4 mM MgCl2, Nuclease-free water, and reaction buffer). Cycling conditions comprised an initial denaturation at 94°C for 3 minutes, followed by 30 cycles of 94°C for 30 seconds, 56°C for 45 seconds, and 72°C for 45 seconds. The cycling was concluded with a final extension step at 72°C for 10 minutes. After the amplification process, amplicons were quantified using the Quant-IT Picogreen dsDNA reagent. A 60 μl aliquot from each amplification was subjected to visualization on a 1.5% agarose gel in TBE buffer (0.5%) to confirm the expected size of PCR-generated amplicons. Nucleic Acid Stain (BioKit) was used to stain the gels, and a 100-base pair DNA ladder (BioKit) served as a size reference. Following gel electrophoresis, the target DNA fragment was excised and purified utilizing the PCR and gel Clean-up kit (Enzo Life Sciences Technology Co., USA). In brief, the DNA fragment was melted in binding DNA buffer (200 μl/100 mg), subjected to wash steps using washing buffer (650 μl), and ultimately eluted in 25 μl of elution buffer. Post-cleanup, samples underwent further visualization through electrophoresis and quantification using the PicoGreen kit. Equivalent concentrations from each sample were then combined into a single pool for subsequent sequencing. A NanoDrop spectrophotometer was employed to assess the cleanliness and DNA content of the pooled samples, ensuring their suitability for downstream sequencing processes. Purification of PCR products was performed using the AmpureXP beads protocol. NGS was outsourced at BGI, Shenzhen, Hong Kong. According to their protocol, library validation was conducted through the Agilent 2100 bioanalyzer (Agilent, USA). Subsequently, the approved libraries underwent 2 × 250 bp paired-end sequencing on the Illumina MiSeq platform (BGI, Shenzhen, China), following Illumina’s standard pipelines.

### 2.5 Bioinformatic workflow

The raw paired-end (PE) fastaq reads were initially demultiplexed, followed by the removal of adapters and primers. Utilizing DADA2 version 1.30.0, an open-source program, the quality assessment, filtering, trimming, and elimination of chimeras were performed on the raw demultiplexed sequences within the R platform version 4.3.2, as outlined (Callahan et al., 2016). The DADA2 pipeline, is distinguished by its ability to cluster identical reads into amplicon sequence variants (ASVs) which exhibit enhanced efficiency and generate fewer erroneous sequences compared to commonly employed pipelines. Employing ASVs for the analysis of the dataset yielded more accurate information regarding composition and diversity compared to conventional operational taxonomic unit (OTU) approaches, wherein sequencing reads are clustered based on a predetermined dissimilarity threshold (Callahan et al., 2017; Needham et al., 2017). In our pipeline, forward and reverse reads obtained from next-generation sequencing underwent trimming and filtering through the ‘filterAndTrim’ function. Subsequently, error rates for trimmed reads were calculated and plotted using the ‘learnErrors’ function, and ASVs were determined utilizing the ‘dada’ function, relying on trimmed reads from all samples. The aligned reads were merged by matching denoised forward reads with the reverse complement of the corresponding denoised reverse reads, thus obtaining complete denoised reads (‘mergePairs’ function). Chimeric sequences were removed through the ‘removeBimeraDenovo’ function. For taxonomy assignment, the resulting list of ASVs from DADA2 was aligned with the reference database available at the Genomic Taxonomy Database (GTDB), as previously described (Parks et al., 2022). Identification of the metabolic pathways was made using Picrust (PICRUSt2 v2.3.0-b, R (v3.4.10), examining three metabolic pathways: KEGG (Kyoto Encyclopedia of Genes and Genomes), Clusters of Orthologous Groups of Proteins (COG), and the MetaCyc metabolic pathway database. The Wilcoxon test and Kruskal-Wallis test were employed to identify differential functions between samples.

### 2.6 Statistical analysis

The standardization of Amplicon Sequence Variants (ASVs) abundance was conducted at a minimum sequencing depth of 39,593 reads, employing the ‘vegan’ package version 2.6.4 (Oksanen J. et al., 2019) in R version 4.3.2. This standardization procedure was implemented to mitigate potential biases arising from variations in sequencing depth. A comprehensive rarefaction analysis, demonstrating the amplification of ASV richness with incremental reads for each sample, was executed utilizing the ‘rarecurve’ function within the ‘vegan’ package. Calculation of alpha diversity indices, including S_obs_, Chao1, and Shannon, was executed through the ‘estimate richness’ function in the ‘phyloseq’ package version 1.46.0 (McMurdie and Holmes, 2013). The graphical representation of these indices was rendered using the ‘ggbetweenstats’ function in the ‘ggstatsplot’ package version 0.12.1 (Patil, 2021). We compared alpha diversity indices between rhizosphere and endosphere using *Welch’s t-test* (Welch, 1947). Beta diversity was assessed with non-metric dimensional scaling (nMDS) based on Bray-Curtis distances, using the ‘plot_ordination’ function in the ‘vegan’ package version 2.6.4 in R version 4.3.2 (Oksanen J. et al., 2019). This analysis aimed to identify differences in microbiota community structure between rhizosphere and endosphere samples. To test dissimilarities in microbial community structure, we used analysis of similarity (ANOSIM with 999 permutations) (CLARKE, 1993). All alpha and beta diversity indices were calculated from standardized ASV abundance. Dominant taxa (representing > 100 reads or at least 0.03% of all sequences) at the phylum and class levels in all samples were visualized using the ‘plot_bar’ function of the ‘phyloseq’ package in R. We considered statistical significance at *P < 0.05*.

## 3 Results

In this research, we used an eDNA-based approach to study the microbiota associated with four desert plants from the Arabian Peninsula namely *H. strobilaceum* (HS), *P. turgidum* (PT), *H. persicum* (HP), and *A. hispidissima* (AH). For each plant, we examined two microbial communities: the soil surrounding and in close proximity to the roots (rhizosphere - R) and the interior of the root tissues (endosphere - E).

### 3.1 Overview of sequencing analysis

**Figure 1:**
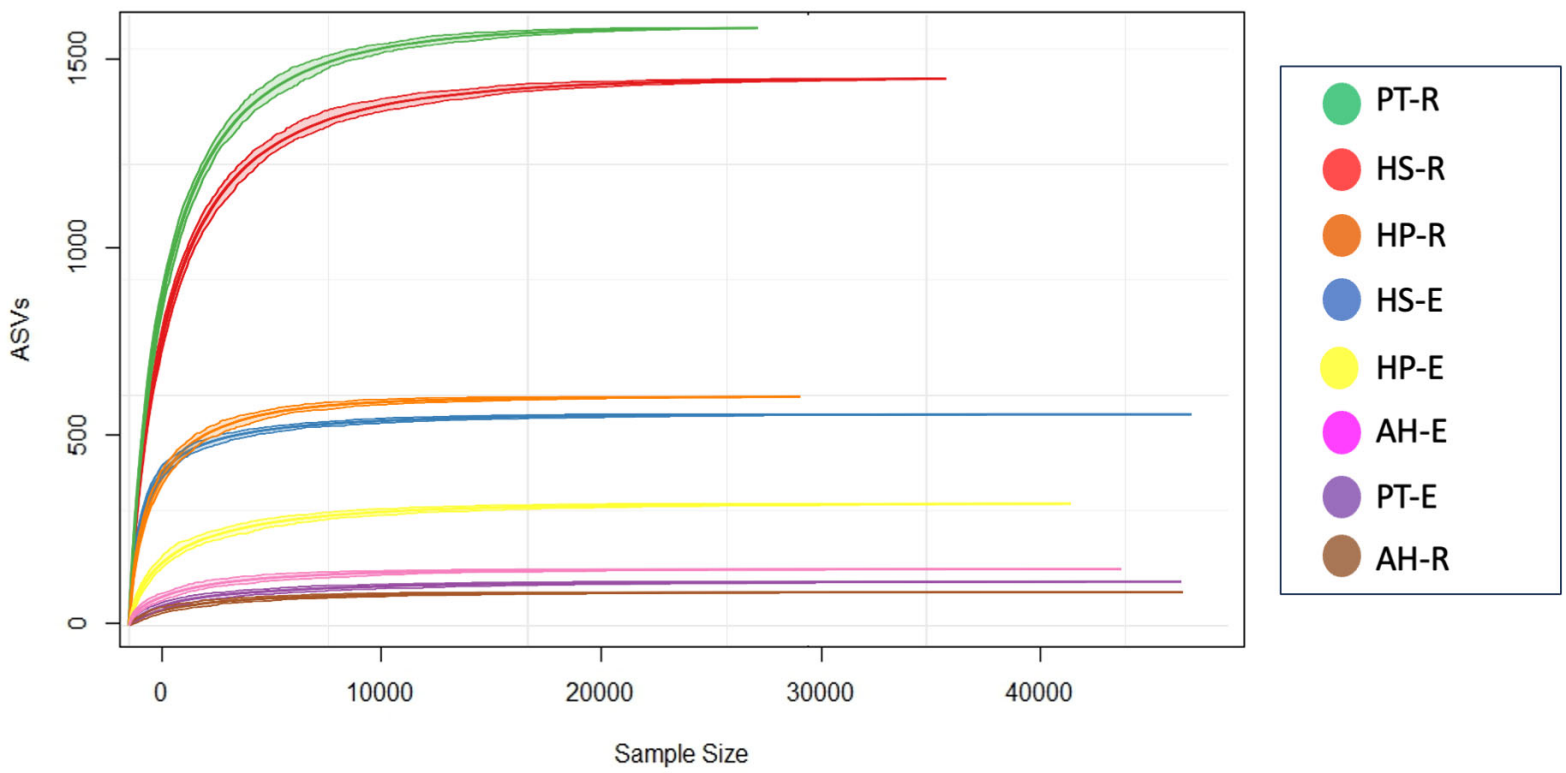
Sequencing analysis based on Amplicon Sequence Variants (ASVs). A rarefaction curve shows that all samples have reached a saturation level indicating that the depth of sequencing is deep enough to capture most microbial composition.

Sequencing generated a total of 542,872 paired-end raw reads across 8 samples, of which 328,894 are merged reads. After the removal of 2,276 potential chimeric reads, we ended up with 310,115 non-chimeric high-quality reads, accounting for 57.12% of the total count, following stringent quality control measures (**Supplementary Table 2**). On average, each sample retained 38,764 qualified reads, with a range from 47,352 to 29,375 reads. In this study, the high-quality reads that have passed the filtering processes were clustered into 4,242 Amplicon Sequence Variants (ASVs) across 8 samples. On average, each sample has 530 ASVs, collectively classified into 39 phyla, 80 classes,189 orders, 343 families, 718 genera, and 806 species. All rarefaction curves exhibited a tendency to reach saturation plateaus, indicating that the sequencing depth employed in this study was sufficient to capture the microbiota community structure (**Figure 1**).

### 3.2 Diversity of the studied microbiota communities

**Figure 2:**
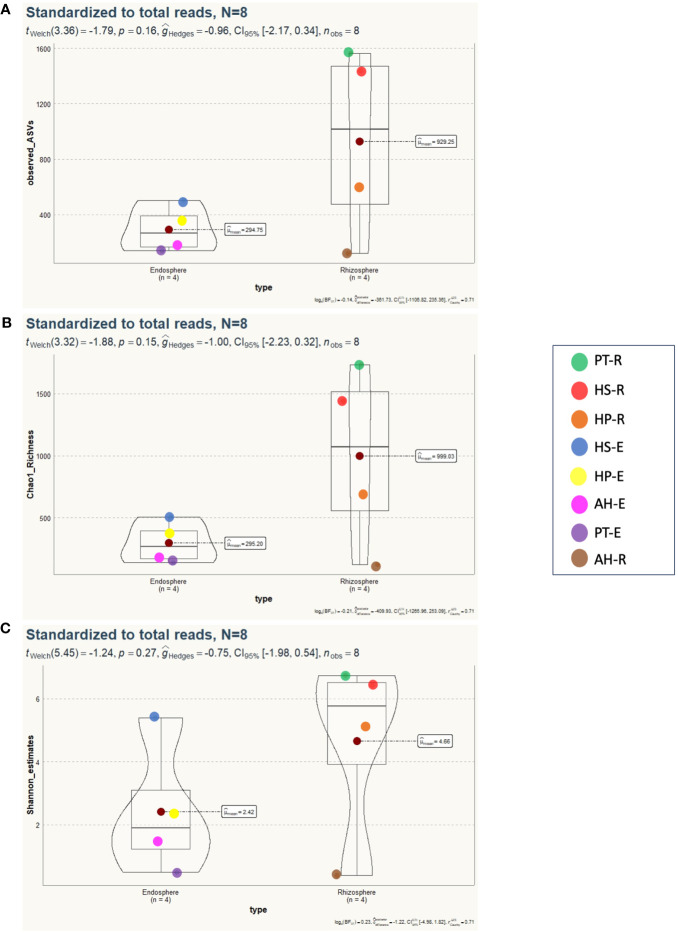
Alpha diversity analysis of 8 microbial communities associated with desert plants. **(A)** Number of observed species (S_obs_) displays the spatial distribution of microbiota diversity, indicating no statistically significant differences between endosphere and rhizosphere microbial communities (*p*=0.16). **(B)** Chao1 richness illustrates a similar trend, with no significant distinctions observed between microbial communities in the rhizosphere or endosphere (*p*=0.15). **(C)** Shannon diversity index reveals no statistically significant differences detected between microbial communities (*p*=0.27).

**Figure 3:**
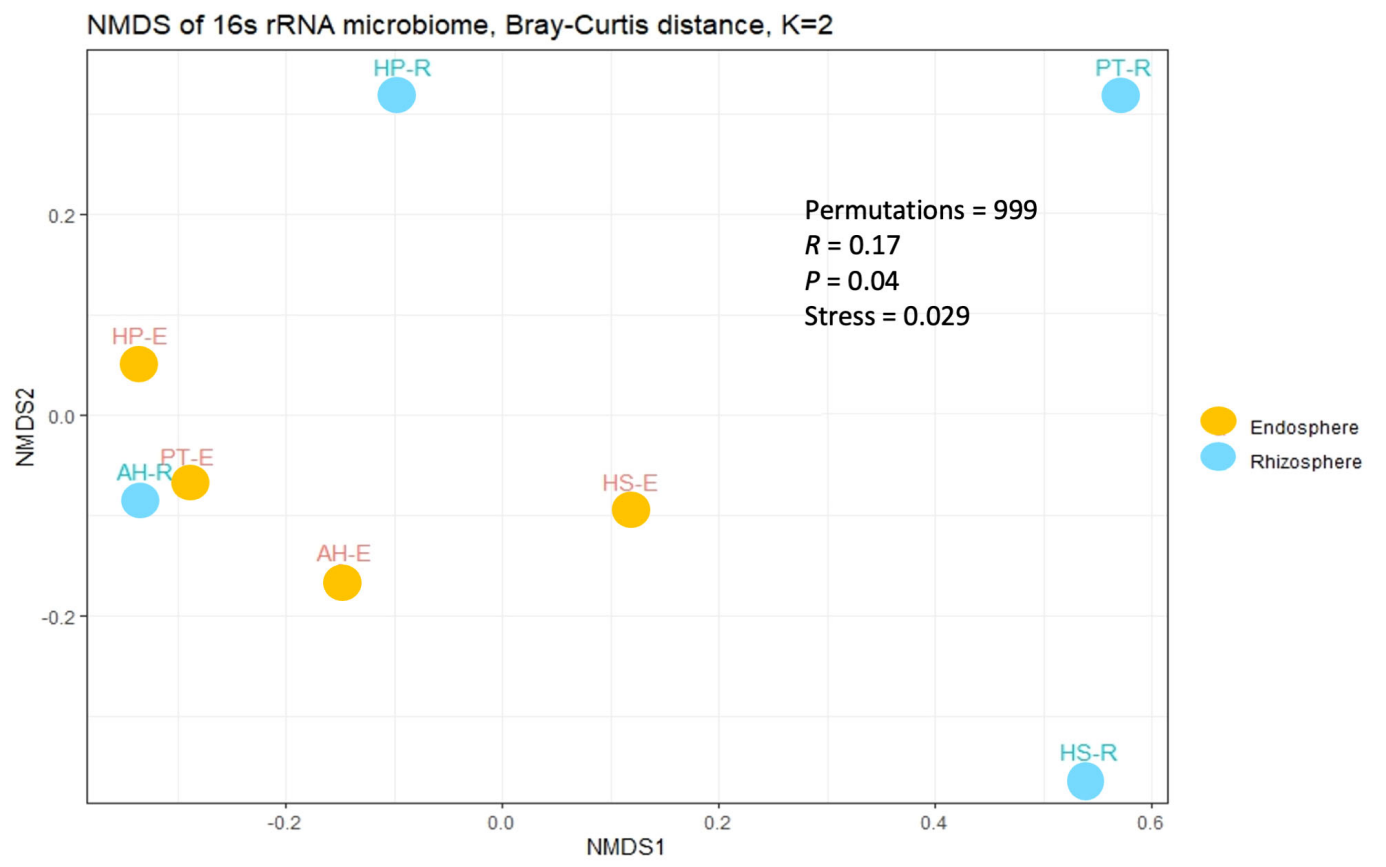
Beta diversity analysis among the studied microbial communities. The non-metric dimensional scaling (nMDS) is based on the Bray-Curtis distance with a stress value of 0.029.

To study the diversity of microorganisms in the eight communities, we analyzed the diversity indices within each sample (α-diversity) and between samples (β-diversity) on normalized datasets with a median sequencing depth of 39,593. Microbiota richness and diversity were evaluated through the observed ASVs (S_obs_), Chao1 index, and Shannon index. All three measures align that the microbiota communities across different samples exhibited relatively varied alpha diversity, as detailed in **Figure 2** and **Supplementary Table 3**. Overall, the rhizosphere communities are more microbial-rich ecosystems than those of the endosphere with an average of 929.25, and 294.75 ASVs, respectively (**Figure 2A**). The Rhizosphere of PT-R and HS-R showed the highest richness (S_obs_) with 1564, and 1438, respectively followed by HP-R with 593 ASVs. While the rhizosphere of AH-R showed the lowest diversity among all samples with 122 ASV. Endosphere communities showed less microbial diversity and ranked based on the number of ASVs as HS-E (504) HP-E (357), AH-E (177), and PT-E (141). This conclusion is consistent with the results of the Chao1 and Shannon indices (**Figures 2B, C**), and the tendency observed in the rarefaction curve (**Figure 1**). In summary, our study reveals that the α-diversity within the studied microbial communities exhibits minimal variation that is not statistically significant, as determined by *Welch’s t-test* (*p > 0.05*). Furthermore, β-diversity analysis was conducted using the non-metric dimensional scaling (nMDS) using Bray-Curtis distances to assess the dissimilarity among microbiota communities of the analyzed samples. The nMDS ordination revealed a distinct separation in microbiota composition grouping patterns between all samples except for AH-R and PT-E which grouped very close to each other (**Figure 3**). It is noteworthy that AH-R and PT-E exhibit the lowest number of ASVs among all samples, with values of 122 and 141, respectively, in contrast to the mean number of ASVs across other samples. This disparity in ASV numbers in AH-R and PT-E potentially influences the overall diversity patterns observed in the dataset. The Analysis of Similarity (ANOSIM) suggests significant dissimilarity between rhizosphere and endosphere microbial communities (permutations = 999, *R = 0.17, p = 0.04*).

### 3.3 Structure and abundance of microbial taxa within the analyzed microbial communities

**Figure 4:**
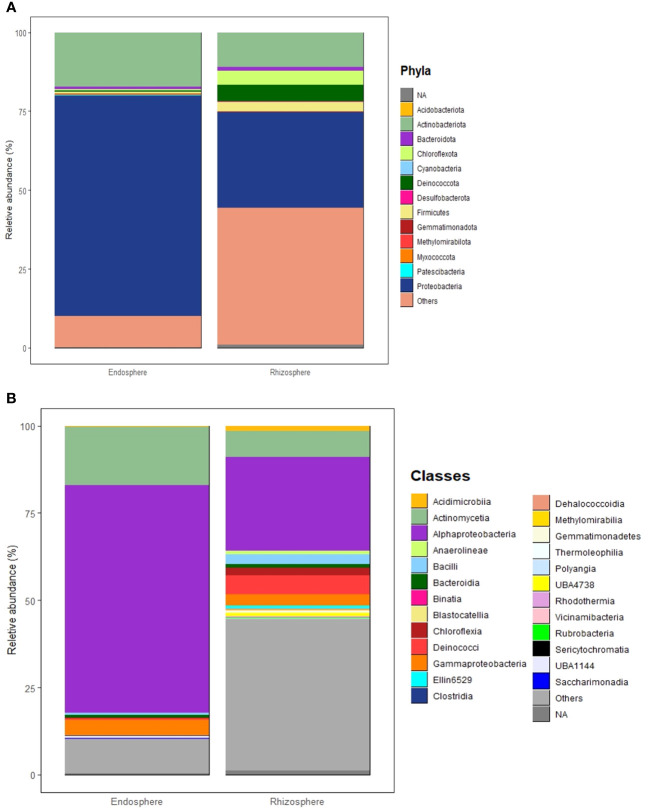
Structure of microbial communities associated with the Rhizosphere and Endosphere of the studied desert plants. **(A)** Most abundant phyla. **(B)** Most abundant classes. Phyla and classes are considered abundant when their total reads exceed 100 and/or a relative abundance of more than 0.03% across all samples.

In this study, most high-quality reads, approximately 99.83%, were identified as bacteria, while approximately 0.14% were assigned to archaea, and 0.02% were categorized as unclassified. Across the eight microbial communities analyzed, a total of 39 microbial phyla and 80 classes were identified. Of these, 13 phyla and 25 classes were considered dominant, contributing to more than 60% of all ASVs across the samples. Dominant taxa were defined as those with a minimum of 100 reads (> 0.03% across all samples) (**Supplementary Tables 4**, **5**). Proteobacteria stands out as the most abundant phylum, making up approximately 53.77% of all high-quality reads (166,753 reads). Following this, Actinobacteria constitutes 14.25% (44,178 reads), Deinococcota 2.2% (7,090 reads), Chloroflexota 2.1% (6,569 reads), Firmicutes 1.6% (4,842 reads), Bacteroidota 1% (3,142 reads), Gemmatiomonadota 0.2% (703 reads), Myxococcota 0.16% (501 reads), Acidobacteriota 0.12% (379 reads), Deslfobacterota 0.08% (250 reads), Methylomirabilota 0.06% (189 reads), Cyanobacteria 0.035% (109 reads), and Patescibacteria 0.032% (100 reads). The most abundant classes overall were Alphaproteobacteria, Actinomycetia, Deinococci, and Bacilli, each showing distinct compositions in each microbial community (**Supplementary Table 5**). In general, endospheric microbial communities are dominated by Proteobacteria, followed by Actinobacteria, Bacteroidota, Firmicutes, Deinococcota, Gemmatiomonadota, Chloroflexota, and Cyanobacteria (**Figure 4A**). On the other hand, the rhizosphere is dominated by Proteobacteria followed by Actinobacteria, Chloroflexota, Deinococcota, Firmicutes, and Bacteroidota (**Figure 4A**). The most prevalent bacterial classes in the endosphere are Alphaproteobacteria, constituting over 50% of the total composition, followed by Actinomycetia and Gammaproteobacteria (**Figure 4B**). Meanwhile, the rhizosphere is dominated by Alphaproteobacteria, accounting for more than 25% of the total composition, followed by Actinomycetia, Deinococci, Gammaproteobacteria, Bacilli, Chloroflexia, Acidomicrobiia, Blastocatellia, Anaerolineae, and Bacteroidia (**Figure 4B**).

### 3.4 Distinct composition of the studied microbial communities

**Figure 5:**
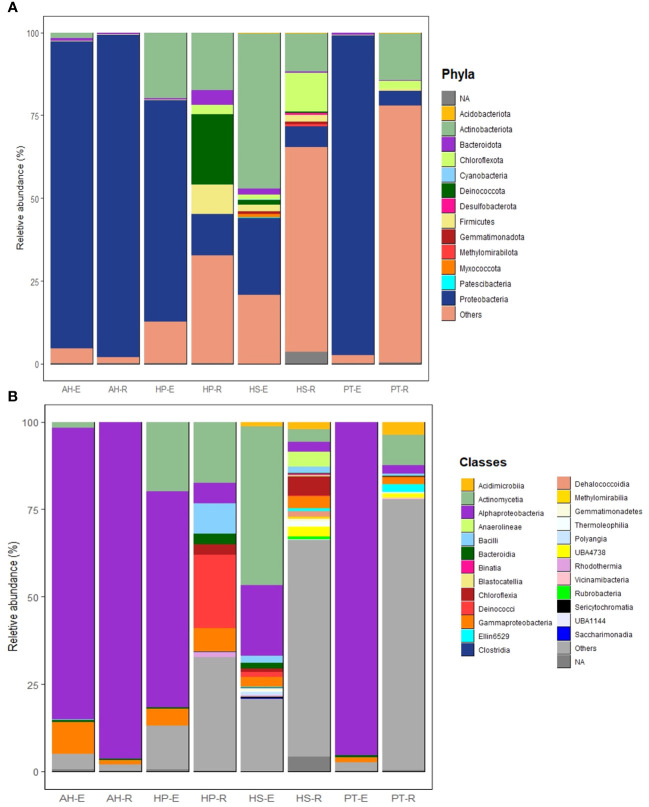
Structure of microbial communities associated with the eight studied microbial communities. **(A)** Most abundant phyla. **(B)** Most abundant classes. Phyla and classes are considered most abundant when their total reads exceed 100 and/or a relative abundance of more than 0.03% across all samples.

The microbial communities in the studied samples showed varying degrees of richness. HP and HS had the most enriched communities followed by PT, while AH exhibited the least varied bacterial composition. In HP, the rhizosphere shows a rich composition characterized by the dominance of Deinococcota (~ 25%) followed by Actinobacteria, Firmicutes, Proteobacteria, Bacteriodota, and Cholorflexota (**Figure 5A**). While its endosphere is mainly composed of Proteobacteria (> 60%), followed by Actinobacteria, Bacteroidota, and Firmicutes. Moreover, the most abundant classes in HP-E are Alphaproteobacteria (> 50%), followed by Actinomycetia, Gammaproteobacteria, and Bacteroidia (**Figure 5B**). Of interest is the prevalence of thermotolerant genera such as *Geodermatophilus*, and radiation-resistant genera such as *Kocuria.* In addition, HP-R contains species adapted to saline soil, such as *Corynebacterium halotolerans* and *Yaniella halotolerans* (**Supplementary Tables 4**, **5**).

The endosphere of HS is dominated by Actinobacteria (~ 48%) and Proteobacteria (~ 25%). HS-E showed a rich composition of various phyla, including Firmicutes, Bacteroidota, Chloroflexota, Deinococcota, Myxococcota, Gemmatiomonadota, Acidobacteria, Cyanobacteria, and Patescibacteria (**Figure 5A**). It’s worth mentioning that Patescibacteria and Cyanobacteria were exclusively detected in HS-E. Additionally, Myxococcota was consistently identified in HS-E, representing ~ 99% of the total presence of this phylum across all samples. The rhizosphere of HS is composed of Chloroflexota, Actinobacteria, Proteobacteria, and Firmicutes. At the class level, HS-E is enriched in Actinomyceita, Alphaproteobacteria, Gammaproteobacteria, Bacilli, Acidomicrobiia, Bacteroidia, Deinococci, Polyangia, and Clostridia. While HS-R is composed of Chloroflexia, Anaerolinea, Gammaproteobacteria, Actinomyceita, Alphaproteobacteria, Bacilli, Dehalococcoidia, Thermoleophilia, and Rubrobacteria (**Figure 5B**). At the genus level, HS is enriched in *Geodermatophilus*, *Trueperaceae*, *Salinicoccaceae*, *Halomonas*, *Mesorhizobium, Fodinicurvata, and Kocuria* (**Supplementary Table 4**). Both the endosphere and rhizosphere microbial communities of HS contain halotolerant bacteria such as *Ornithinicoccus halotolerant* and *Nocardioides halotolerans*.

PT-E is dominated by Proteobacteria (> 90%) with minor representation of Bacteroidota, Firmicutes, and Acidobacteria. While PT-R is dominated by Actinobacteria followed by Proteobacteria, Chloroflexota, Acidobacteria, and Firmicutes (**Figure 5A**). The most abundant classes in PT-E include Alphaproteobacteria, Gammaproteobacteria, and Bacteroidia, while PT-R is composed of Actinomycetes, Acidimicrobiia, Alphaproteobacteria, Gammaproteobacteria, and Bacilli (**Figure 5B**). PT-R is enriched in radiotolerant bacteria such as *Kocuria* sp., and *Rubrobacter radiotolerans.* In addition, PT-R shows enrichment in thermotolerant bacteria such as *Quasibacillus thermotolerans*.

AH exhibits the least diverse microbial community, with both the rhizosphere and endosphere predominantly composed of Proteobacteria (over 90%), with minor representations of Actinobacteria, Bacteroidota, and Firmicutes (**Figure 5A**). The most abundant classes in both AH-E and AH-R included Alphaproteobacteria, Gammaproteobacteria, and Bacteroidia (**Figure 5B**).

### 3.5 Genomic functional prediction of active metabolic pathways in the studied desert microbial communities

**Figure 6:**
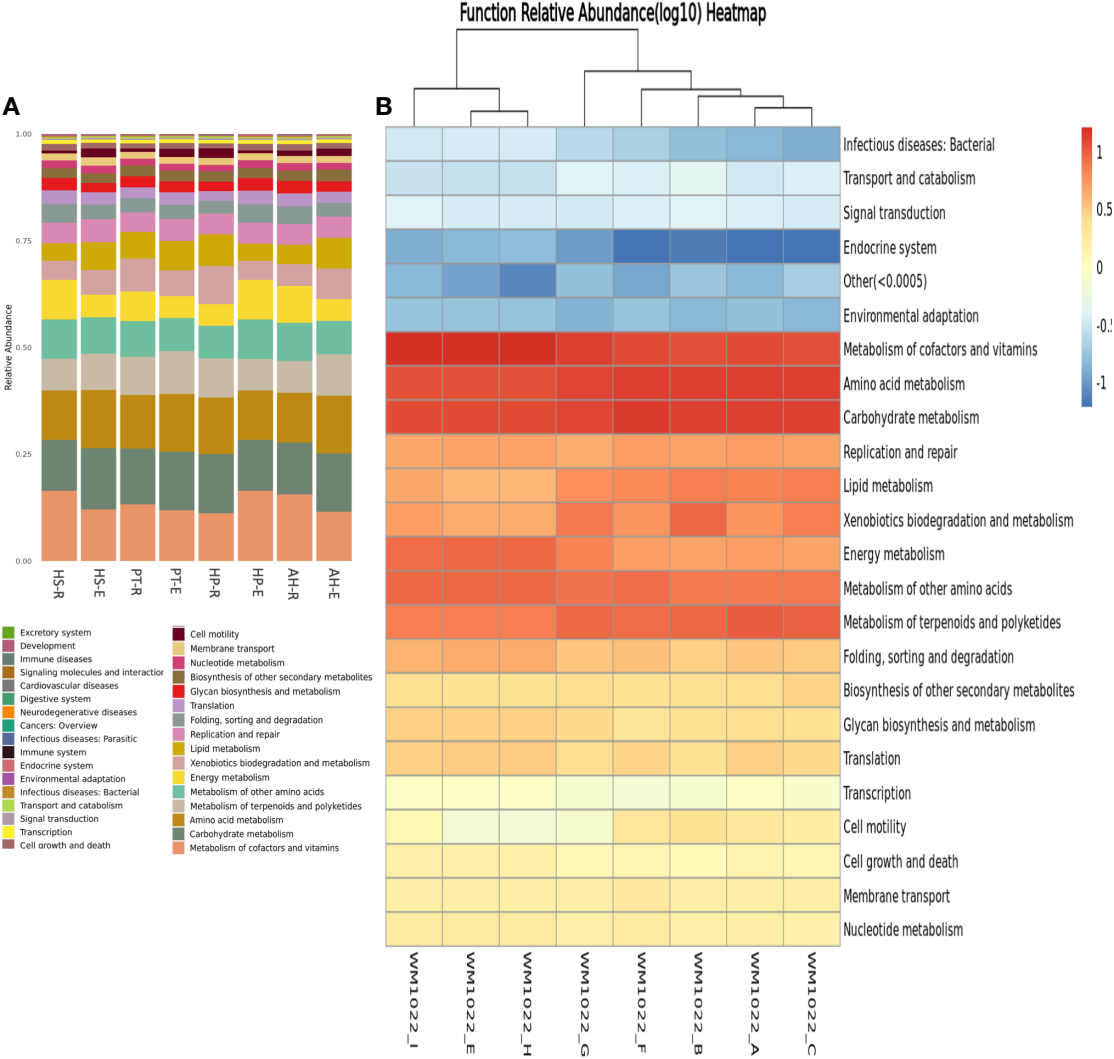
KEGG genomic functional analysis of analyzed microbiome samples. **(A)** Boxplot shows the relative distribution of predicted metabolic functions within each sample and when compared to one another. **(B)** Heatmap of predicted functions where Longitudinal clusters refer to a functional similarity and horizontal clustering refers to functional similarity within different samples. Functional between samples are more similar with closer distances or shorter branching. The color depth of each module in the functional heat map reflects the varying richness of specific groups of functional genes. Relative abundance values are log-transformed for normalization.

For the examination of active metabolic pathways within the microbial communities associated with desert plants, genomic functional assessments were conducted using KEGG (**Figure 6**), MetaCyc, and GOS (**Supplementary Figures 2**, **3**).

A consistent metabolic pattern, characterized by active metabolism, is observed across all samples, highlighting the metabolic versatility and richness within the microbial communities in the desert ecosystem. The most abundant KEGG pathways are associated with the metabolism of carbohydrates, amino acids, terpenoids, polyketides, vitamins, glycan, and xenobiotics. Then succeeded by cell motility, glycan synthesis, and secondary metabolite biosynthesis, indicating the robust metabolic activity of these microbial communities (**Figure 6**). This result aligns with MetaCyc genomic prediction which shows enrichment in genes related to the metabolism of carbohydrates, lipids, amino acids, and nucleotides. In addition, there is an enrichment in genes related to transcription, translation, and protein transport (**Supplementary Figure 2**). Of note is the higher abundance of genes related to posttranslational modifications, protein turnover over, and chaperons’ synthesis which might serve an ecological role to protect from heat stress in the desert environment. There is enrichment also in defense mechanisms which increase the resilience and competency of microbes in harsh ecosystems. Utilizing GOC analysis to categorize the functions of potential genes reveals that predominant functions are consistently shared across all samples, comprising biosynthesis of vitamins, carbohydrates, amino acids, fatty acids, and lipids, in addition to cell structure biosynthesis and carbohydrate degradation (**Supplementary Figure 3**).

## 4 Discussion

Desert ecosystems harbor a crucial reservoir of microbial diversity, although they remain largely understudied. These microorganisms contribute significantly to ecological stability and biogeochemical cycles in their habitats (Alsharif et al., 2020; Weber et al., 2015). Understanding the biodiversity, compositions, and functions of desert microbial communities is crucial for gaining insights into global changes and identifying potential threats and opportunities applicable to agricultural ecosystems with climate change. In this study, we utilized an eDNA metagenomic approach to uncover the structure, diversity, and potential function of rhizosphere and endosphere microbial communities associated with four plants native to the Arabian Peninsula desert. Our findings indicate that rhizosphere communities exhibit greater richness in diverse microbial taxa as opposed to the endosphere, which aligns with prior studies (Schlaeppi et al., 2014; Behairi et al., 2022). The composition of the rhizosphere microbial community is predominantly influenced by soil properties and climatic zones, with a lesser impact from the host plant genotype (Xu et al., 2023; Zhang et al., 2022). Conversely, microorganisms that infiltrate and colonize plant tissues are challenged with more stringent selection criteria controlled by host-related factors such as genotypes, age, tissue type, metabolism, and immunity (Dastogeer et al., 2018; Dastogeer et al., 2020). Moreover, endosphere microbial communities must manipulate both the host and pre-existing microbial populations to successfully establish colonization (Dastogeer et al., 2020).

### 4.1 The unique microbial composition of desert microbiota contributes to their resilience

Our findings revealed that the predominant phyla in the studied communities are Proteobacteria and Actinobacteria. These results are consistent with the majority of the reports on the microbiota of the Arabian Peninsula desert (Inostroza et al., 2017; Bang et al., 2018; Andrés-Barrao et al., 2017; Eida et al., 2018; Guesmi et al., 2022; Li et al., 2023; Osman et al., 2016). Bacteria of these phyla serve crucial ecological roles to their host plants. For example, Proteobacteria is metabolically versatile and plays roles in nutrient cycling and organic matter decomposition (Zhang et al., 2019; Rath et al., 2019). Within this phylum, common genera like *Rhizobium*, *Mesorhizobium*, *Sinorhizobium*, and *Azorhizobium* are known to plant symbionts with nitrogen-fixing capabilities (Pini et al., 2011). Additionally, Actinobacteria members exhibit strong versatility, thriving under extreme conditions such as salinity, pH variations, low water availability, extreme temperatures, and intense radiation (Mohammadipanah and Wink, 2016). Actinobacteria encompasses diverse genera such as *Streptomyces*, *Saccharomonospora*, and *Nocardioides* (Nafis et al., 2019).

Among the four studied plants, *H. persicum (HP)*, and *H. strobilaceum* (HS) have the most enriched microbial communities, followed by *P. turgidum* (PT), whereas *A. hispidissima* (AH) exhibited the least diverse microbial composition. To the best of our knowledge, our study is the first report on the AH microbiome while there are some previous reports on the microbiome of PT, HP, and HS, with a focus on rhizosphere microbes (Guesmi et al., 2022; Li et al., 2023).

*H. persicum* (HP) is a desert plant known for its ability to withstand drought (Yang and Lv, 2023). Our study revealed that the endosphere of HP is dominated by Proteobacteria and Actinobacteria. In contrast, the rhizosphere exhibits a more diverse microbial composition, with Deinococcota being the dominant phylum. Previous research reported that the rhizosphere of HP is mainly composed of Actinobacteria, Chloroflexi, and Bacteroidetes (Li et al., 2023). Interestingly, HP is also rich in Archaea, such as Thaumarchaeota, and fungi (Li et al., 2023). Consistent with the literature (Khan and Khan, 2020), our data reveals that HP is enriched in radiation-resistant, thermotolerant, and halotolerant taxa.

*H. strobilaceum* (HS) is a desert halophyte known for its resilience to both salinity and drought, with reported instances of this plant thriving in tidal zones along coastlines (Marasco et al., 2016).. Our data indicates that HS hosts a diverse microbe, characterized by the prevalence of Actinobacteria in the endosphere and Cholorflexota in the rhizosphere. HS is characterized by an enrichment in halotolerant taxa. In line with our findings, earlier reports demonstrated the abundance of Halotolerant genera such as *Halomonas*, *Mesorhizobium*, *Fodinicurvata*, and *Salegentibacter* in the rhizosphere of HS (Li et al., 2018; Marasco et al., 2016). In another study, the isolation of 414 strains from both the rhizosphere and endosphere of HS showed the prevalence of Proteobacteria, followed by Firmicutes, Actinobacteria, and Bacteroidetes. This was accompanied by an enrichment in halotolerant taxa, particularly within the *Halomonas* genus (Behairi et al., 2022).

*P. turgidum* (PT) is a desert xerophyte known for its ability to withstand drought conditions (Abd Allah et al., 2019). The endosphere of PT is dominated by Proteobacteria, whereas the rhizosphere is characterized by a diverse composition with Actinobacteria, Proteobacteria, and Chloroflexota as the dominant taxa. In a prior study, it was observed that PT is predominantly populated by Actinobacteria and Proteobacteria (Eida et al., 2018). The study also isolated species from dominant genera including *Bacillus*, *Rhizobium*, *Kocuria*, *Microbacterium*, and *Pseudomonas* (Eida et al., 2018). Our data reveals that the rhizosphere of *P. turgidum* (PT) is enriched in radiotolerant, halotolerant, and thermotolerant bacteria. This aligns with a previous study, which cultured various radiation-resistant species from PT, such as *Promicromonospora panici*, *Kocuria rhizophila*, *Micrococcus* sp., and *Microbacterium* sp (Guesmi et al., 2022).. In the same study, researchers conducted metagenomic analysis to examine the bacterial community of irradiated roots. The results demonstrated a shift in microbial structure characterized by an increased abundance of Actinobacteria and Proteobacteria. Interestingly, this shift was accompanied by the induction of metabolic pathways related to oxidative stress tolerance, including DNA repair (Guesmi et al., 2022).

### 4.2 The Arabian Peninsula desert exhibit habitat-specific microbial communities

The abundance of metabolism-related genes is consistently high across all samples, consistent with previous reports (Ronca et al., 2015). This active metabolism serves an ecological function, enhancing the resilience of these communities as they adapt to desert conditions and contributing to the support of associated plants. Of note is the enrichment in pathways related to DNA transcription, protein synthesis, posttranslational modification, and chaperone biosynthesis. These findings suggest an adaptation to fast protein turnover and high heat stress, emphasizing the plant’s resilience in the challenging desert environment (Guisbert et al., 2004; Kim et al., 2021). Overall, genes associated with carbohydrate and amino acid metabolism are consistently abundant across all samples, underscoring the biological significance of functions like metabolism and the rapid growth of microorganisms. Carbohydrate decomposition serves as an energy source for microbial growth and development, particularly in the context of limited nutrient availability such as desert conditions. Additionally, amino acid catabolism plays a crucial role in providing nutrition to the entire microbial community by breaking down proteins into smaller amino acid molecules. This functional analysis is aligned with the abundance of microbes contributing to nutrient cycling. For example, the Acidobacteriota phylum which was mostly detected in the rhizosphere of PT, plays a crucial role in nutrient cycles (Kristensen et al., 2021), drought resistance (Huber et al., 2022b), nitrogen cycling via reduction of nitrate to nitrite (Huber et al., 2017), and plant growth-promotion (Yoneda et al., 2021). Acidobacteria has also been reported in harsh conditions such as acidic mine drainage (Huber et al., 2022a; Falagán et al., 2017), and rocks (Marnocha and Dixon, 2014). Additionally, the widespread presence of the Thermoleophilia class is known to exert a significant influence on microbial communities, particularly in arid deserts with limited fertilizer availability compared to agricultural ecosystems (Cui et al., 2018). Nitrogen-fixing microbes, specifically from the genera *Rhizobium* and *Geodermatophilus* were found to be prevalent across all samples.

Besides, our data reveals enrichment of desert microbiome with adaptable microbes that exhibit resilience to harsh conditions such as high temperatures, intense radiation, salinity, and limited nutrients. For example, thermotolerant bacteria, such as Thermomicrobia, a class within the Phylum Chloroflexota (formerly Chloroflexi) were widespread in the studied samples. These microbes are characterized by their hyper-thermophilic nature and their ability to survive and thrive in extremely high temperatures of up to 65-75°C while exhibiting high nitrate-oxidizing activity (Sorokin et al., 2014). Some species from this class have been isolated from extreme ecosystems, including the flank of Kilauea Volcano (Hawai’i) (King and King, 2014) and geothermal soil in Waitike New Zealand (Houghton et al., 2015). Additionally, our findings highlight the presence of microbes with radiation resistance, exemplified by genera Geodermatophilus and Deinococcus, known for their unique ability to resist gamma radiation. Species such as *Deinococcus radiotolerans* (Cha et al., 2014) in the rhizosphere of HS and *Rubrobacter radiotolerans* (Egas et al., 2014) in the rhizosphere of PT showcase remarkable resistance to gamma and UV radiation (Egas et al., 2014; Verma et al., 2017). The *Trueperaceae* genus, belonging to the Deinococcota phylum, was identified in the rhizosphere microbial communities of HP and HS, with a lesser presence in PT. This genus is notable for its exceptional resistance to ionizing radiation and thermotolerance (Albuquerque et al., 2005). Furthermore, the desert microbiome includes species capable of surviving high salinity, with notable examples like *Corynebacterium halotolerans* (Chen et al., 2004) and *Yaniella halotolerans* (Nouioui et al., 2018; Chen et al., 2010) in the rhizosphere of HP, *Nocardioides halotolerans* (Dastager et al., 2008), and *Ornithinicoccus halotolerans* (Zhang et al., 2016) associated with the microbial communities of HS. These halotolerant species exhibit tolerance to varying levels of salinity, reflecting their adaptation to the challenging desert habitat.

## 5 Conclusion

Our study sheds light on the intricate microbial diversity and functional potential within the Arabian Peninsula desert, emphasizing the adaptability and resilience of these communities to extreme environmental conditions. The inclusion of metabolically active, radiation, heat, drought, and salinity-tolerant microbes underscores the ecological significance of these microorganisms. These findings contribute to a deeper understanding of desert ecosystems and pave the way for potential applications, including their role in enhancing agricultural resilience amid climate change and global warming. Future investigations, including targeted culturing of strains with traits needed to enhance the productivity of heat and drought-susceptible crops, alongside biological profiling *in vitro* since the functional annotation using PICRUSt2 does not distinguish strain-specific functionality (Douglas et al., 2020). In addition, in planta assessment will further elucidate the practical implications of these microbial communities for broader ecological and agricultural contexts.

## Data availability statement

The datasets presented in this study can be found in online repositories. The names of the repository/repositories and accession number(s) can be found in the article/**Supplementary Material**.

## Author contributions

WM: Conceptualization, Data curation, Methodology, Writing – original draft, Writing – review & editing, Funding acquisition. TA-I: Conceptualization, Data curation, Writing – review & editing, Methodology. AS-T: Formal analysis, Visualization, Writing – review & editing.

## Supplementary material

Supplementary Table 1 GPS coordinates for sites of sample collection in this study

Supplementary Table 2 Summary of the bioinformatic analysis using DADA2 pipeline for all samples

Supplementary Table 3 Detailed values for diversity indices.

Supplementary Table 4 All 16S rRNA sequences data and taxonomy information of microbial communities for the studied samples.

Supplementary Table 5 Details of the most abundant taxa in the study

Supplementary Figure 1 Representative photos and description of the desert plants in the study.

Supplementary Figure 2 MetaCyc genomic functional analysis of analyzed microbiome samples. **(A)** Heatmap of predicted functions where Longitudinal clusters refer to a functional similarity and horizontal clustering refers to functional similarity within different samples. Functional between samples are more similar with closer distances or shorter branching. Relative abundance values are log-transformed for normalization. **(B)** Boxplot shows the relative distribution of predicted metabolic functions within each sample and when compared to one another.

Supplementary Figure 3 GOS genomic functional analysis of analyzed microbiome samples. **(A)** Heatmap of predicted functions where Longitudinal clusters refer to a functional similarity and horizontal clustering refers to functional similarity within different samples. Functional between samples are more similar with closer distances or shorter branching. Relative abundance values are log-transformed for normalization. **(B)** Boxplot shows the relative distribution of predicted metabolic functions within each sample.
