# Supplementary figures and images for: High-throughput sequencing reveals the structure and metabolic resilience of desert microbiome confronting climate change
Source: Front Plant Sci. 2024 Mar 5;15:1294173. doi: 10.3389/fpls.2024.1294173 (PMC10953687; doi:10.3389/fpls.2024.1294173)

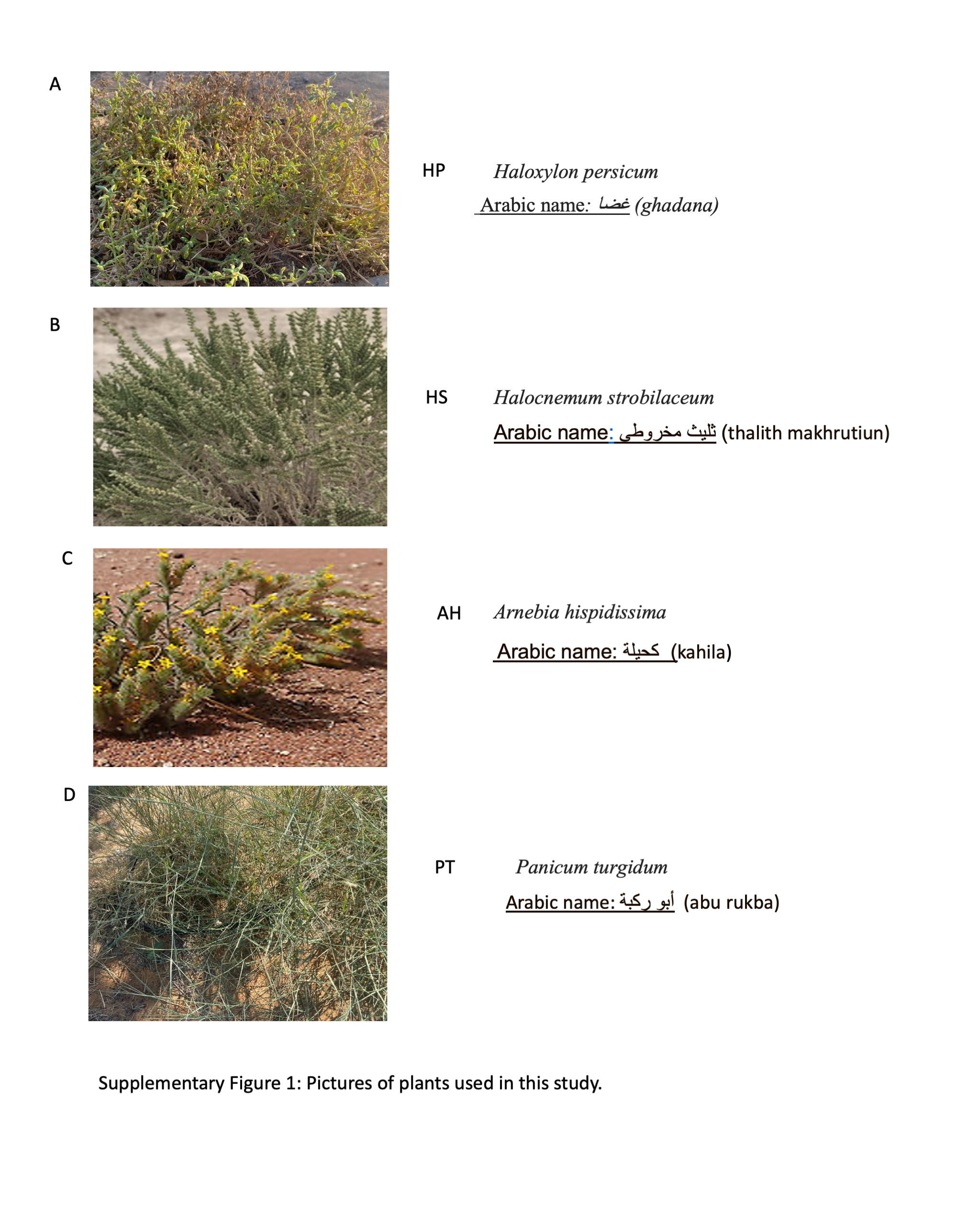

Supplement: Supplementary Figure 1 — Representative photos and description of the desert plants in the study. [file Image_1.tiff]

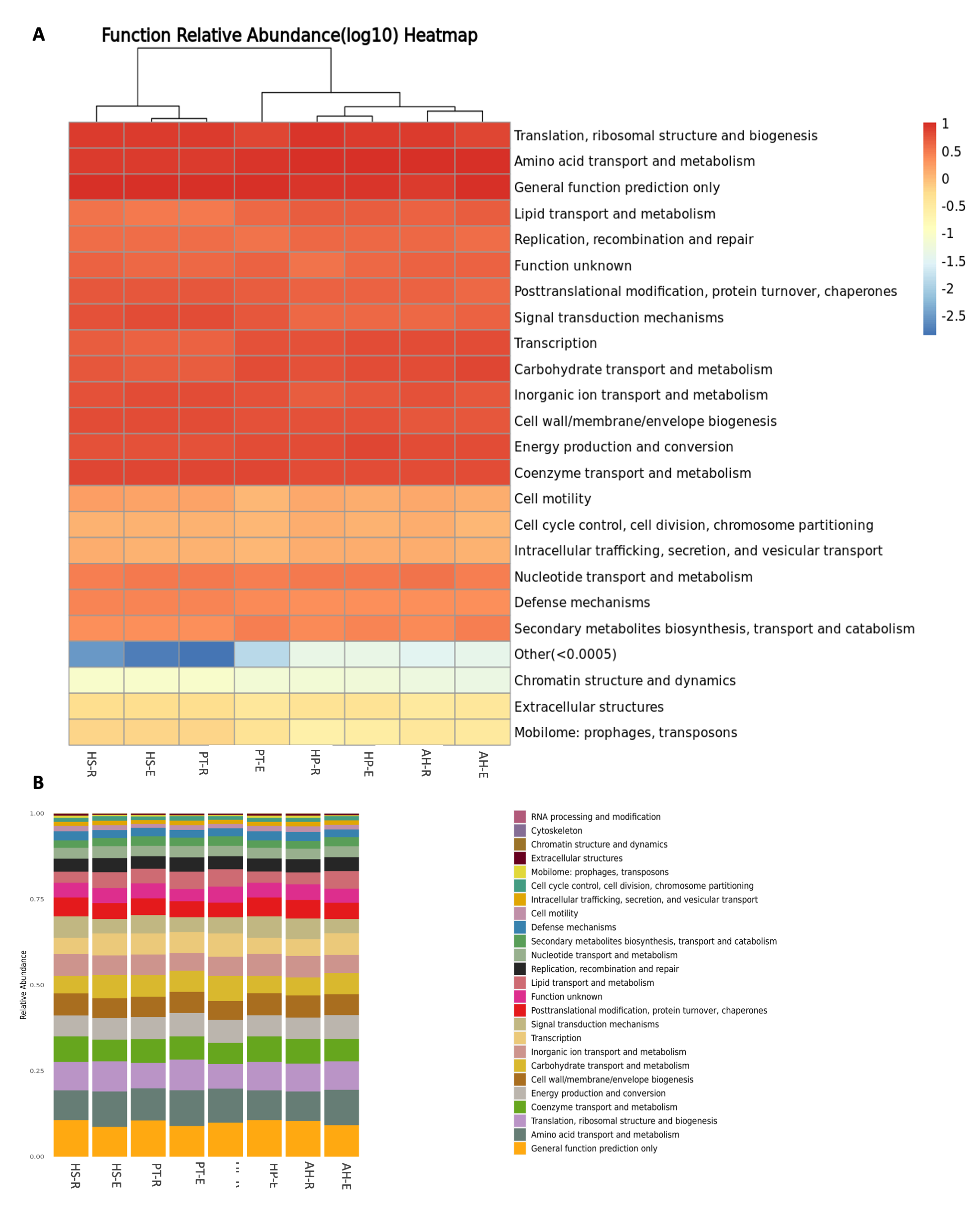

Supplement: Supplementary Figure 2 — MetaCyc genomic functional analysis of analyzed microbiome samples. (A) Heatmap of predicted functions where Longitudinal clusters refer to a functional similarity and horizontal clustering refers to functional similarity within different samples. Functional between samples are more similar with closer distances or shorter branching. Relative abundance values are log-transformed for normalization. (B) Boxplot shows the relative distribution of predicted metabolic functions within each sample and when compared to one another. [file Image_2.tiff]

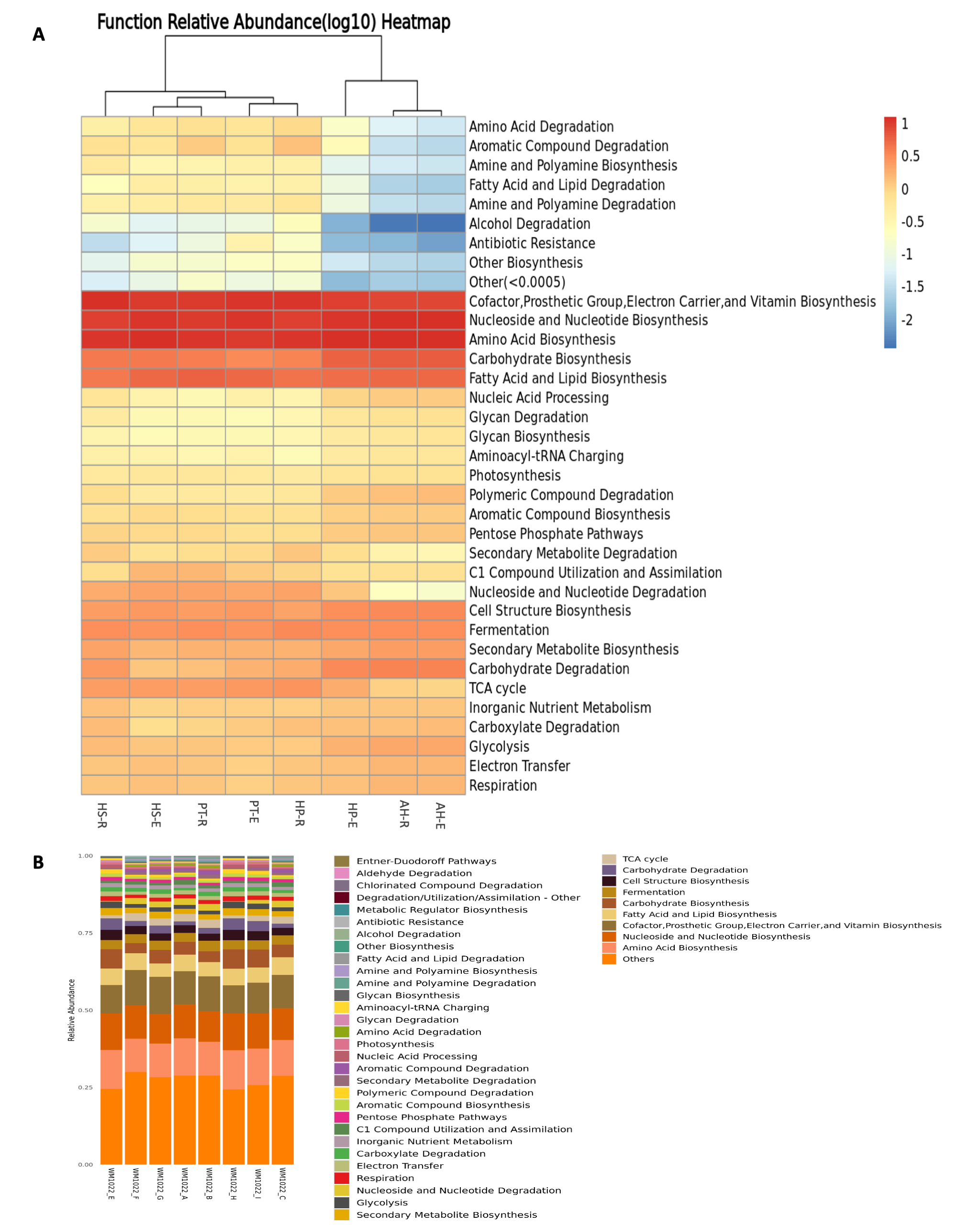

Supplement: Supplementary Figure 3 — GOS genomic functional analysis of analyzed microbiome samples. (A) Heatmap of predicted functions where Longitudinal clusters refer to a functional similarity and horizontal clustering refers to functional similarity within different samples. Functional between samples are more similar with closer distances or shorter branching. Relative abundance values are log-transformed for normalization. (B) Boxplot shows the relative distribution of predicted metabolic functions within each sample. [file Image_3.tiff]
